# Congenital Cataracts – Facial Dysmorphism – Neuropathy

**DOI:** 10.1186/1750-1172-1-32

**Published:** 2006-08-29

**Authors:** Luba Kalaydjieva

**Affiliations:** 1 Western Australian Institute for Medical Research and Centre for Medical Research, The University of Western Australia, Hospital Avenue, WA 6009 Nedlands, Australia

## Abstract

Congenital Cataracts Facial Dysmorphism Neuropathy (CCFDN) syndrome is a complex developmental disorder of autosomal recessive inheritance. To date, CCFDN has been found to occur exclusively in patients of Roma (Gypsy) ethnicity; over 100 patients have been diagnosed. Developmental abnormalities include congenital cataracts and microcorneae, primary hypomyelination of the peripheral nervous system, impaired physical growth, delayed early motor and intellectual development, mild facial dysmorphism and hypogonadism. Para-infectious rhabdomyolysis is a serious complication reported in an increasing number of patients. During general anaesthesia, patients with CCFDN require careful monitoring as they have an elevated risk of complications. CCFDN is a genetically homogeneous condition in which all patients are homozygous for the same ancestral mutation in the *CTDP1 *gene. Diagnosis is clinical and is supported by electrophysiological and brain imaging studies. The major differential diagnosis is Marinesco-Sjögren syndrome. The definitive diagnosis is molecular, based on homozygosity for the *CTDP1 *mutation. *CTDP1 *maps to 18qter and encodes a protein phosphatase whose only known substrate is the phosphorylated serine residues of the carboxy-terminal domain of the largest subunit of RNA polymerase II, indicating that CCFDN affects basic cellular processes of gene expression and developmental regulation. Families benefit from genetic counselling and predictive testing. Management includes surgical treatment of the cataracts, and rehabilitation and corrective orthopaedic surgery for the peripheral neuropathy. Thus, the most disabling manifestations, though not curable, are manageable, and allow an acceptable quality of life and everyday living. Current data indicate that patients survive well into adulthood.

## Disease name

Congenital Cataracts Facial Dysmorphism Neuropathy (CCFDN) syndrome

## Definition/diagnostic criteria

CCFDN is a complex developmental disorder of autosomal recessive inheritance which, so far, has been found to occur exclusively in patients of Roma (Gypsy) ethnicity [1,2].

### Primary diagnostic criteria

• Bilateral congenital cataracts, microcornea, microphthalmos, micropupils

• Developmental delay

• Small stature and low weight

• Hypo/demyelinating neuropathy

• Mild facial dysmorphism

• Mild hypogonadism

### Additional criteria

• Mild cognitive deficit

• Cerebral and spinal cord atrophy on neuroimaging

• Post-infectious rhabdomyolysis

• Osteoporosis

## Epidemiology

The CCFDN mutation is particularly common among the Rudari, an endogamous, recently founded Roma population, in whom the carrier rate is around 6–7% [3,4]. In other Gypsy groups, it is ~1%. The disorder has so far been diagnosed in over 100 Roma patients, whose countries of residence reflect past and recent migrations of the affected population, as well as access to healthcare and awareness of this syndrome among health care providers. Most known affected subjects belong to the traditional Roma population of Balkan and Central European countries (Bulgaria, Romania, Serbia, Greece, Hungary, the Czech Republic), or are recent migrants to Austria and Germany; with cases also diagnosed in Italy, France, The Netherlands, USA and Canada. CCFDN should be considered as the most likely diagnosis in infants of Roma ethnicity presenting with congenital cataracts.

## Clinical description

The CCFDN phenotype is complex and affects multiple organs and tissues, mostly during intra-uterine development. Detailed descriptions of the clinical manifestations are available [2,5-7].

Prenatal eye development is invariably affected and the congenital cataracts are the first CCFDN manifestation, recognisable in early infancy. The cataracts are bilateral and are characterised by anterior or posterior cortical dense opacities with clouding of the adjacent part of the lens nucleus or total cataracts involving the whole lens. These are accompanied by microcornea, microphthalmos and micropupils. Additional ophthalmological features include floppy eyelids and long and dense eyelashes, nystagmus and strabismus [7].

Early motor, as well as intellectual, development is delayed, with most patients starting to walk between 2 and 3 years and to talk after 3 years of age.

On physical examination, all patients are of small stature and most are also of low weight. Head circumference is reduced; however, its ratio to height is normal. Facial dysmorphism develops in late childhood and is more evident in male than in female subjects. It includes a prominent midface with a well developed nose, thickening of the perioral tissues, forwardly directed anterior dentition and hypognathism. Skeletal deformities are present in most patients, especially adults. Foot deformities, namely pes cavus or pes equinovarus with clawing of the toes, are very common. Claw hand deformity develops in the course of the disease and is observed in most adult patients. Kyphoscoliosis may develop and leads to reduced vital capacity.

The most striking neurological manifestation is a symmetric, distally accentuated, predominantly motor peripheral neuropathy. Motor involvement is evident in all affected subjects, including young children, and progresses to severe disability in the third decade. Loss or depression of tendon reflexes, initially in the lower limbs, is universally observed. Nerve conduction velocity is normal in early infancy and declines after age 18 months, stabilizing at ~20 m/s after 4 years [8]. Distal motor latency is increased. Sensory action potential values are of normal amplitude, suggesting a relatively uniform degree of slowing of conduction across nerve fibres, consistent with congenital hypomyelination. Neuropathological evidence of primary hypomyelination is provided by nerve biopsy studies, showing also a superimposed demyelination/remyelination and axonal degeneration increasing with age.

Other, less consistent, neurological abnormalities include bilaterally extensor plantar responses, choreiform movements, mild ataxia and upper limb postural tremor. Abnormal electroencephalographic recordings are a very common finding, with usually diffuse slow wave activity. Brain and spinal cord abnormalities have been identified in many patients by magnetic resonance imaging (MRI) examination [2]. The changes appear to be age-related and may not be detectable in affected children. The abnormalities include diffuse cerebral atrophy with enlargement of the lateral ventricles, and spinal cord atrophy sometimes also involving the medulla oblongata. A recent study, using diffusion tensor MRI in a small group of CCFDN patients revealed a higher apparent diffusion coefficient and lower fractional anisotropy in the vermis and medulla oblongata, suggesting axonal loss in these parts of the brain [8]. Mild non-progressive cognitive deficit is often present; however, its assessment should take into account the visual impairment, as well as possible social deprivation of the affected subjects.

Severe myalgia, weakness and massive myoglobinuria, presumably following unspecified viral infections, are reported in an increasing number of patients. Para-infectious rhabdomyolysis, which is rare in the general population [9], may be a common environmentally-triggered feature of the CCFDN syndrome, possibly related to an inability of the transcription machinery to meet the higher demands imposed by the febrile condition. Patients and care-providers should be aware of this complication and seek prompt medical attention to prevent acute kidney failure.

Sex hormone (testosterone and oestrogen) levels are decreased. External secondary sexual characteristics appear normal; however most adult female patients have irregular menstrual cycles progressing to secondary amenorrhea at age 25–35 years. Bone mineral density is reduced both in compact and trabecular bone.

## Aetiology

CCFDN is a genetically homogeneous condition, where all patients are homozygous for the same ancestral mutation. The *CCFDN *gene maps to 18qter [1]. The recently identified mutation is a C>T substitution 389 bp downstream of the exon 6/intron 6 junction of the *Carboxy-Terminal Domain Phosphatase 1 (CTDP1) *gene [3]. The mutation occurs in an antisense Alu element and triggers a rare mechanism of aberrant splicing: it creates a perfect donor splice site which, in turn, activates an upstream cryptic acceptor site, leading to the insertion of 95 nucleotides of the intronic Alu sequence into the processed *CTDP1 *transcript. The insertion causes a shift in the reading frame and a premature termination codon. Since both normal and aberrant splicing occur in CCFDN cells [3], the mutation is predicted to lead to partial protein deficiency. The *CTDP1 *gene encodes a protein phosphatase, commonly referred to as FCP1, whose only known substrate is the phosphorylated serine residues of the carboxy-terminal domain (CTD) of the largest subunit of RNA polymerase II [10]. RNA polymerase II is responsible for the transcription of protein-encoding genes in eukaryotic cells, and the level of CTD phosphorylation is considered to be a key mechanism for the regulation of gene expression [11]. In addition to, and independent of its enzyme activity, FCP1 has been proposed to play other roles in transcription: as a positive transcription regulator, an elongation factor, a factor counteracting the Srb-10 complex (which down-regulates the expression of cell cycle and developmentally regulated genes), and in the mechanisms of splicing. At this stage, it is unclear which molecular function(s) is most affected by the *CCFDN *mutation and thus responsible for the disease phenotype.

Regardless of the need for understanding the specific molecular pathogenesis, it is already clear that CCFDN affects basic cellular mechanisms of gene expression and their developmental regulation. The disorder belongs to the (currently) small group of "transcription syndromes" and, at present, is the only known defect directly involving RNA polymerase II-mediated gene expression.

## Diagnostic methods

The clinical diagnosis is based on observation of the symptoms and manifestations described above, supported by electrophysiological and brain imaging studies. The definitive diagnosis is molecular, based on homozygosity for the splicing mutation in intron 6 of the *CTDP1 *gene. The test is a polymerase chain reaction (PCR)-based restriction fragment length polymorphism (RFLP) assay, relying on the abolition of an *NlaIII *restriction site by the mutation [3]. Molecular testing based on linkage analysis may be problematic, due to the recent origin of the mutation on a common haplotype background and the persisting presence of the ancestral haplotype on normal chromosomes in the same population [3].

So far, CCFDN is known to affect patients of Roma (Gypsy) ethnicity, with the history of this population helping to identify the molecular basis of the disease. It is logical to expect that there are affected individuals of different ethnicity displaying similar phenotypic features and that these patients would have other mutations of the same gene. In such cases, sequencing analysis of *CTDP1*, as well as of the *SIL1 *gene recently found to be mutated in Marinesco-Sjögren syndrome (MSS) [12,13], will not only help the diagnostic investigations in the specific affected families, but will also contribute to understanding the molecular pathogenesis of the two disorders. It is advisable to make cultured cells (skin fibroblasts, lymphoblastoid cells, myoblasts, or Schwann cells) available for molecular studies, since the mutation(s) can be predicted to cause partial deficiency, and are thus likely to occur in introns and regulatory sequences and to require RNA-based analysis for elucidation.

## Differential diagnosis

In early infancy, when most features of the CCFDN syndrome have yet to appear, a differential diagnosis to be considered is galactokinase deficiency, an inborn error of metabolism common among the Roma which, if untreated, leads to the development of cataracts in the first weeks of life [14,15]. The two conditions can be distinguished by specialised ophthalmological examination, searching for the additional ocular manifestations of CCFDN (described above), as well as by testing for the two founder mutations – in the *CTDP1 *and *GALK1 *genes.

The major differential diagnosis is MSS [12,13,16,17], whose phenotypic features partly overlap those of CCFDN [6,8] (Table 1). While substantial clinical heterogeneity exists among patients diagnostically labelled as MSS, mutations in the *SIL1 *gene on chromosome 5q seem to result in a relatively homogeneous phenotype, characterised by congenital cataracts, developmental delay, severe ataxia and cerebellar atrophy, and chronic myopathy [12,13]. The heterogeneity is still incompletely resolved, with some "typical", as well as "atypical" MSS cases failing to show linkage to 5q and/or mutations in *SIL1 *[13,18].

**Table 1:** Clinical criteria in differential diagnosis of Congenital Cataracts Facial Dysmorphism Neuropathy (CCFDN) and Marinesco-Sjögren syndrome (MSS)

**SYMPTOMS**	**CCFDN**	**MSS**
Microcornea, microphthalmos, micropupils	Yes	No
Developmental delay	Yes	Yes
Small stature	Yes	Yes
Facial dysmorphism	Yes, mild, late childhood	No
Peripheral neuropathy	Yes, hypomyelinating	Rare, variable type
Ataxia	Rare, mild*	Severe cerebellar
Brain MRI	Cerebral atrophy*	Cerebellar atrophy
Myopathy	Acute para-infectious rhabdomyolysis*	Chronic
Cognitive deficit	Mild*	Variable degree
Hypogonadism	Mild*	Variable in degree and origin (hypo/hypergonadotrophic)

* Manifestations whose frequency and nature require further characterisation.

## Genetic counselling

The disorder is autosomal recessive, with a Mendelian risk of 25% for subsequent pregnancies. Given the high frequency of the mutation, and the endogamous tradition of the affected population, discussions of consanguinity are irrelevant and can be counterproductive. Most affected families are very receptive to genetic counselling and predictive testing.

## Prenatal diagnosis

Prenatal analysis is best performed by testing for the disease mutation on DNA extracted from chorionic villus samples.

## Management

Management includes the treatment of cataracts, and rehabilitation and corrective surgery for the peripheral neuropathy. Eye surgery may be complicated by strong postoperative inflammatory reactions, as well as a strong unspecific foreign-body reaction to contact lenses and intra-ocular lenses, with a generally better tolerance to intra-ocular lenses [7]. CCFDN patients are prone to developing severe and potentially life-threatening complications during anaesthesia, such as pulmonary oedema, inspiratory stridor, malignant hyperthermia and epileptic seizures [6,7] and thus need close monitoring and possibly intensive post-operative care.

## Prognosis

The recent recognition of CCFDN as a novel disease entity, and the lack of prospective follow-up does not allow an evidence-based prognosis regarding life expectancy. Current observations indicate that patients survive well into adulthood (the oldest known cases being over 40 years of age). While the most disabling manifestations involving the eyes and the peripheral nervous system are not curable, they are manageable, allowing an acceptable quality of life and performance of daily activities.

## Unresolved questions

### Clinical features

The nature of the pathological changes in the central nervous system is still to be resolved. At present, it is unclear whether the brain and spinal cord atrophy observed on MRI result from primary hypomyelination, as is the case for the peripheral nervous system [2,5] or secondary degenerative processes. Furthermore, the recently reported diffusion tensor MRI evidence of axonal loss in the brain [8] needs to be confirmed in larger groups of patients. The nature of the endocrine abnormalities is also poorly defined. Although the hypogonadism seen in CCFDN patients has been classified as hypogonadotrophic [2], the evidence is not conclusive, and the problem may in fact reside in changes in the expression profile of the gonads. The frequency of rhabdomyolysis in CCFDN patients and the nature of the factors triggering this severe complication remain to be determined; careful collection of case histories and patient follow-up will provide this information.

### Molecular mechanisms

Further studies are needed to elucidate the specific molecular processes that lead to the phenotypic manifestations of CCFDN. The mutated protein is believed to be of universal importance for the regulation of RNA polymerase II activity and the *CCFDN *mutation is expressed in all cell types investigated, yet the clinical phenotype is limited to just a few of them. This cell specificity, possibly resulting from differences in back-up mechanisms and in the relative contribution of other CTD phosphatases to RNA polymerase II regulation, is a major question, resolution of which will not only help understanding of the disease itself, but could also contribute to unravelling fundamental mechanisms of the regulation of gene expression.
